# Probable Sudden Unexpected Death in Dogs With Epilepsy (pSUDED)

**DOI:** 10.3389/fvets.2021.600307

**Published:** 2021-04-27

**Authors:** Enrice Huenerfauth, Jasmin Nessler, Johannes Erath, Andrea Tipold

**Affiliations:** Department of Small Animal Medicine and Surgery, University of Veterinary Medicine, Hannover, Germany

**Keywords:** sudden death, canine, idiopathic epilepsy, pSUDED, (p)SUDEP

## Abstract

Sudden unexpected death in human epileptic patients (SUDEP) is defined as death related to recurrent unprovoked seizures, death occurring unexpectedly, and suddenly in a patient with reasonable state of health, without an obvious medical cause of death, trauma, asphyxia, or intractable status epilepticus, and in post mortem examination no obvious reason for death can be found. “Probable SUDEP” (pSUDEP) is defined as SUDEP not confirmed pathologically. The adapted abbreviation for dogs is used in the following: “pSUDED” (probable sudden unexpected death in dogs with epilepsy). The aim of the present monocentric retrospective study using an online questionnaire was to evaluate the occurrence of pSUDED. Data of canine patients presented with seizures between 01/1998 and 05/2018 were retrospectively analyzed and classified according to their etiology (*n* = 1,503). Owners were contacted by telephone to participate in answering a validated questionnaire. A total of 509 owners were reached, and 373 owners completed the questionnaire. In addition to signalement (e.g., breed), special attention was paid to the frequency and presentation of seizures and seizures in the context of death. Fifty-one percent (191/373) of the dogs were dead at the endpoint of the study. A large proportion of the dogs was euthanized (149/191) because of seizure severity or health problems unrelated to seizures. Idiopathic epilepsy (IE) was diagnosed in 19/34 dogs which died unexpectedly. Of these seven animals had to be excluded for further investigation of pSUDED because of status epilepticus or aspiration pneumonia as a result of the seizures. In 12 dogs with IE the last seizure event occurred between 6 h and ~3 months before death. pSUDED was suspected in these dogs and an occurrence rate of 4.5–10% was calculated. pSUDED appears in a similar occurrence rate as human SUDEP and should be considered as a possible complication in epileptic dogs. The results of this study suggest that dogs with IE but especially those with brachycephalic syndrome and cluster seizures have an increased risk to die of pSUDED. Owners of dogs with seizures should be educated about the risk of sudden death in dogs with epilepsy.

## Introduction

There is a 1.6–3-fold increase in standardized mortality rate (SMR) in human patients with epilepsy compared to the general population (1). In human medicine, the risk for sudden unexpected death in epilepsy patients (SUDEP) was estimated to be 7–12% up to 17% of all deaths in patients suffering from epilepsy and occurs especially after generalized tonic-clonic seizures (GTCS) (2–6).

In human medicine, autopsies are performed to establish the cause of “unexpected” death or to clarify the underlying disease (7, 8). **“Definite SUDEP”** is defined in human medicine as “sudden unexpected, witnessed or unwitnessed, non-traumatic death occurring in benign circumstances in an individual with epilepsy with or without evidence for a preceding seizure and excluding a documented status epilepticus. The postmortem examination does not reveal the cause of death” (9). An exclusion criterion for SUDEP is a preceding status epilepticus with a seizure activity duration of more than 30 min (9, 10). A **probable SUDEP** (pSUDEP) lacks postmortem examination but complies with definite SUDEP: “unexpected death in a reasonable state of health, during normal activities and in benign circumstances and without a known structural cause of death” (9). In the majority of SUDEP, death of patients is unwitnessed and the occurrence of preceding seizures is unknown (9). Potential risk factors include among others: seizure frequency, long duration of epilepsy, amount of antiepileptic drugs (AED) applied, and development of mental disability (11). Furthermore, Hughes reports in his review that subtherapeutic AED levels are a risk factor for SUDEP (12).

The majority of SUDEP cases occurred in direct relation with or up to 1 h after GTCS (4). Lhatoo et al. depicted some cases where SUDEP occurred 3–5 days after the last GTCS in hospitalized, monitored patients (13).

Around 0.6% of the dog population and up to 18.3% of dogs of specific breeds are affected by epilepsy (14–16). There are several studies about quality of life (QoL) in canine patients with epilepsy (17–20), but sudden unexpected death in dogs is only sporadically reported and poorly studied (21–24). Idiopathic epilepsy (IE) is described as an exclusion diagnosis for dogs of a certain age group with recurrent seizures without an underlying structural cause, but with a (presumed) genetic influence (25, 26).

The described high occurrence rate of SUDEP in people (2) and the occurrence of single canine cases of probable sudden unexpected death in dogs with epilepsy (pSUDED) in our clinic aroused our interest. The purpose of this study was to detect the occurrence rate of pSUDED in dogs with IE and to evaluate if pSUDED is a relevant risk factor for death in dogs with IE. In addition, factors influencing pSUDED, such as the number of applied AEDs, seizure frequency, or breed, and brachycephalic syndrome (BCS) should be evaluated. Thus, we hoped that the analysis would allow us to educate and advise dog owners and to adapt the therapy and seizure management accordingly in the future.

## Materials and Methods

### Data Search

For the retrospective monocentric study, patient data such as signalment, diagnostic results, and diagnosis were collected using the software easyVet (IFS Informationsysteme GmbH, Hannover, Germany) and Anidata (Comitas Software GmbH, Leipzig, Germany) of the Department of Small Animal Medicine and Surgery of the University of Veterinary Medicine Hannover Foundation. The following key words were used to perform the search: seizure, epilepsy, status epilepticus.

Included were 1,503 dogs with recurrent (one and more) seizures, which were presented to the University of Veterinary Medicine Hannover between 01/1998 and 05/2018. Further inclusion criteria were at least one general and one neurological examination and available results of blood tests for further analysis, as well as urine test if available. According to their diagnosis the cases were divided into pSUDED study population (idiopathic epilepsy) and into the control group (structural epilepsy and reactive seizures). To diagnose IE findings had to be unremarkable [TIER I, International Veterinary Epilepsy Task Force (27)].

**Figure 1:**
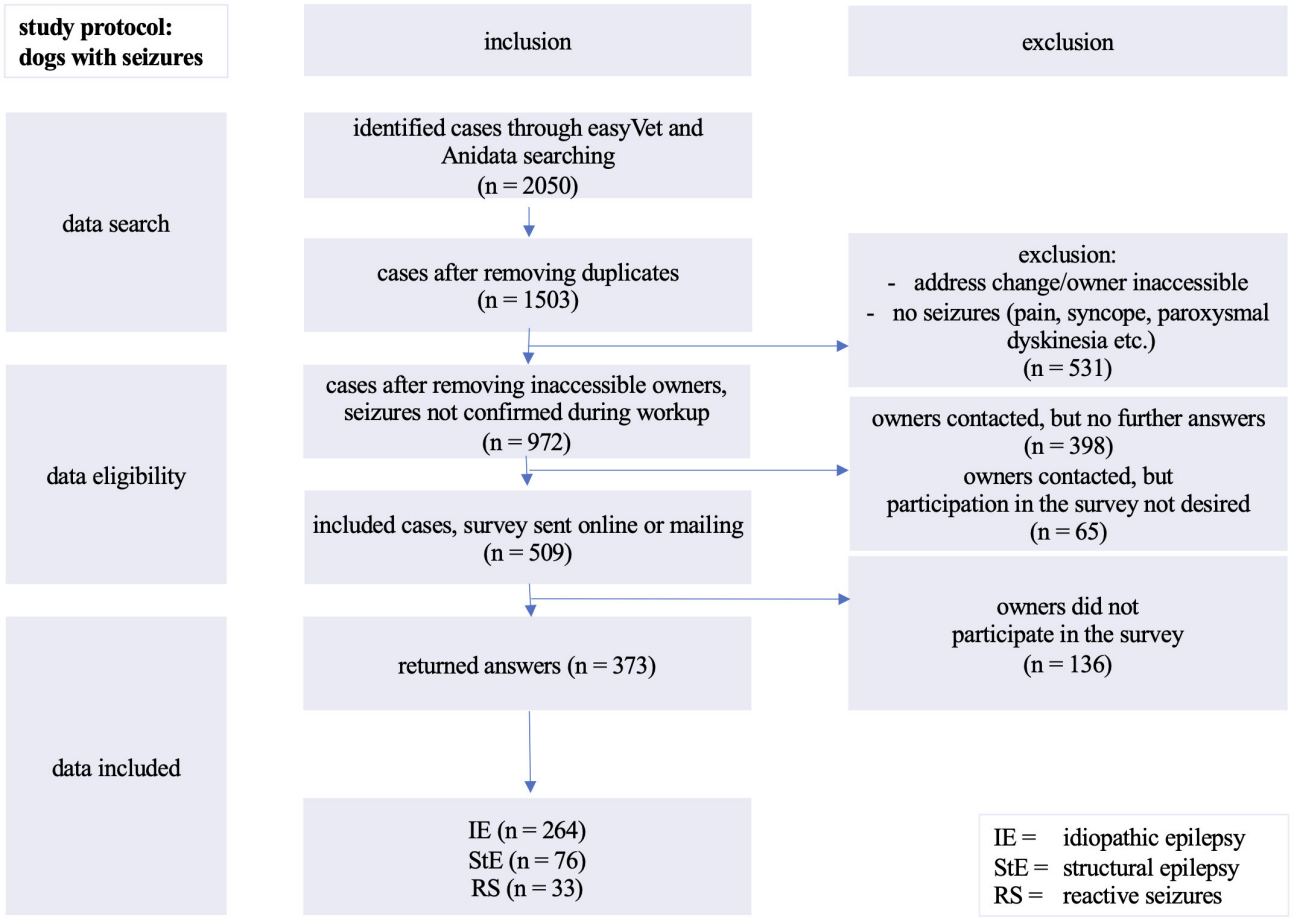
Inclusion of evaluated cases: analyzed cases of dogs with seizures presented between 01/1998 and 05/2018 at the Department of Small Animal Medicine and Surgery, University of Veterinary Medicine in Hannover, Germany.

Cases had to be excluded due to the impossibility to contact the owners for further information and clinical signs not reflecting epileptic seizures during diagnostic approach (Figure 1). The control cases with structural epilepsy and reactive seizures were excluded for the analysis of pSUDED but evaluated comparatively for the potential life expectancy of different groups with seizures (26).

Contacted owners who gave their consent to participate received an access link to the online survey by e-mail or in printed form by post. In some cases, owners preferred a telephone interview to answer the standardized online questionnaire. The survey was performed according to the General Data Protection Regulation and with permission of the data protection officer of the University.

### Study Design/Questionnaire

For the current study a standardized online questionnaire was used. Some of the questions were adopted from a validated questionnaire (17), and additional questions were developed to evaluate death in dogs with epilepsy. These additional questions were validated in a small survey and reviewed by Diplomates of the European College of Veterinary Neurology (AT, JN) and employees of center for E-Learning, didactics, and educational research (ZELDA) (FE) regarding structure, phrasing, understanding, and processing of the questions.

The agreement between the results of the survey and the description of the disease allows us to conclude a constructed validity (17). The online survey was done using the online application LimeSurvey and data was collected with the help of LimeSurvey GmbH (an open source survey tool/LimeSurvey GmbH, Hamburg, Germany; http://www.limesurvey.org). The questionnaire was adapted according to whether the dog was alive or dead, categorized by a “yes or no” first question. The survey was constructed in several parts and included 71 questions for 6 subject areas (Supplementary Survey):

- Signalement and any underlying disease (breed, sex, age and date of birth, weight, questions about a traumatic brain injury)
- Seizure history (e.g., age at onset of the first seizure, diagnostic examinations, diagnosis, cluster seizures, status epilepticus)
- Seizure severity and frequency
- Antiepileptic drugs
- Quality of life
- Causes of death (deceased or euthanized), death under or without observation, sudden unexpected death, death during a seizure and its presentation and duration (cluster seizures or status epilepticus), as well as the time interval between the last seizure and death, euthanasia in relation with epilepsy, or due to other circumstances.

### Study Population

The diagnosis for inclusion of dogs with idiopathic epilepsy or controls was reached on the basis of the definitions of the International Veterinary Epilepsy Task Force (26, 27).

To evaluate the frequency of occurrence of pSUDEDs, only dogs with the diagnosis of IE TIER I or II, were selected (26). pSUDED was defined according to the criteria published by Nashef et al. (9). By definition, any underlying cause that could have a direct influence on death lead to exclusion of the patient for analysis of pSUDED cases (9).

According to previous classifications of breeds with BCS (28–30), the following breeds have been designated as such: Chihuahua, French and English Bulldog, Pug Dog, Dogue de Bordeaux, Boston Terrier, Boxer, Boxer Bulldog Mix, Bulldog Mix, Bullmastiff Boxer Mix, Cavalier King Charles Spaniel, Continental Bulldog, Pekinese, and Shih Tzu.

### Statistical Analysis

For statistical analysis Microsoft^®^ Excel 2019 was used and descriptive statistics were performed to get an overview of the average age and life expectancy after the onset of the disease. The occurrence rate of pSUDED cases in this study population was calculated and compared with the percentage of all deceased dogs with IE presented to our clinic in the last 20 years.

The influence of gender and breed on pSUDED was analyzed by calculating odds ratios. Multivariable regression analysis was used as a method to investigate relationship between independent variables and one outcome. Two independent variables, cluster seizures and status epilepticus, as well as seizure frequency, breed, and seizure type were compared and examined for association with pSUDED. The ANOVA, part of the regression statistics, shows the relevance of the coefficient table. The statistically significant variable in multivariable regression was defined as *p* < 0.05. Chi-square tests consider correlations of established variables in comparative groups and were performed to compare the number of applied AEDs in pSUDED cases to other dogs with IE.

## Results

About one third (531/1,503) of the detected cases had to be excluded due to change of owner's address and/or telephone contact or due to clinical signs not related to epileptic seizures during the diagnostic procedure (Figure 1).

Almost another third (463/1,503) of potential participants could not be reached by telephone. In 65 out of 463 cases the dog owners did not want to participate in the survey. A total of 509 dog owners could be contacted by telephone or via e-mail.

The participation rate was 73.3% and yielded the absolute number of 373 completed surveys eligible for further analysis. One quarter of the 509 surveys (26.7%; *n* = 136) were not completed or returned.

**Figure 2:**
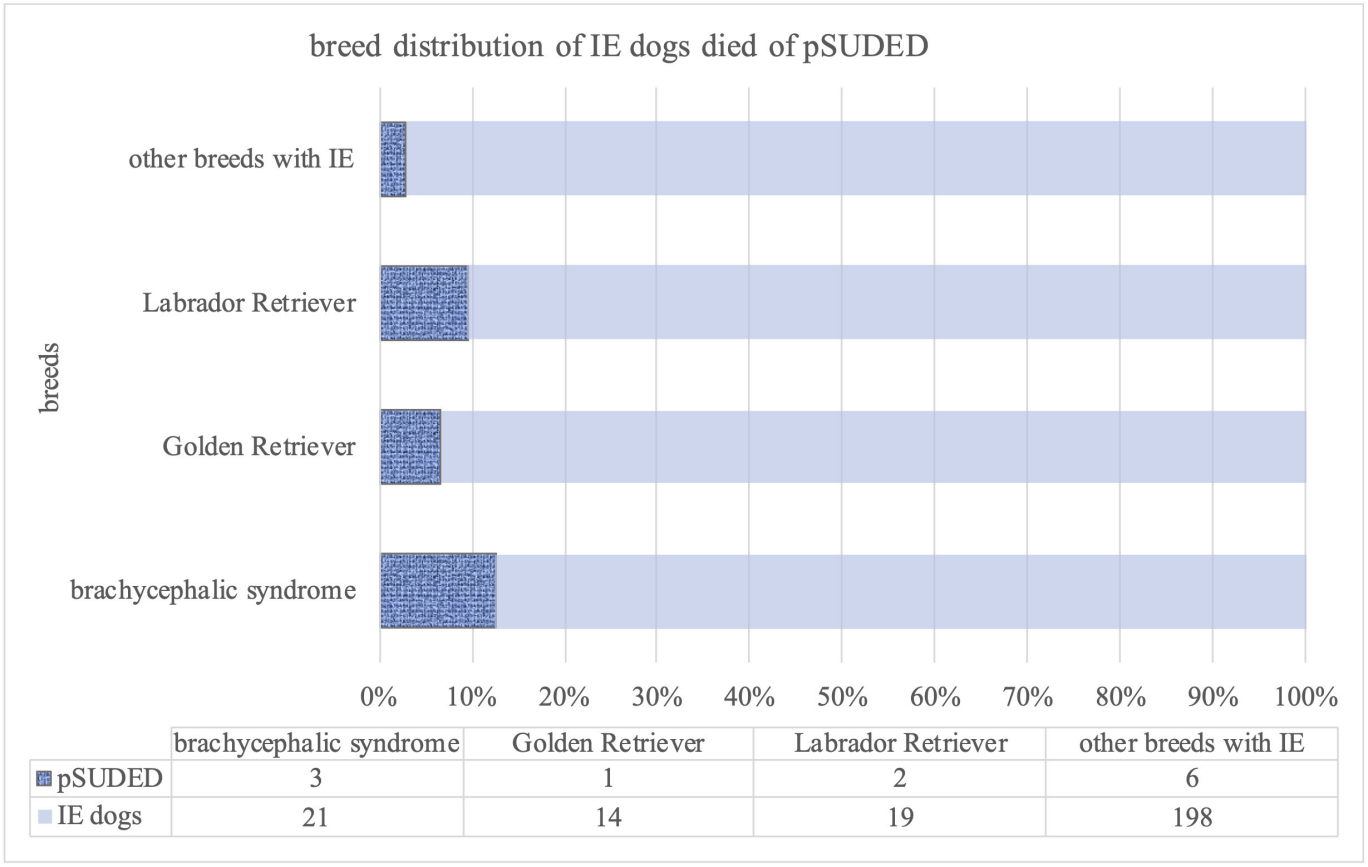
Graphical representation of included dogs with seizures.

The study population included 264 dogs (167 males, 97 females) of different breeds with suspected or diagnosed IE (TIER I and TIER II confidence level) (27). Additionally, 76 dogs with diagnosed or suspected structural epilepsy (42 males, 34 females) and 33 dogs (16 males, 17 females) displaying reactive seizures were evaluated only for analysis of life expectancy for the control group (Figure 2).

For the evaluation, 264 dogs with IE were included (Figure 1). Of these, 54.54% (144/264) were still alive at the time of the study. One hundred and twenty dogs with IE (45.4%; 120/264) died or were euthanized (Figure 2).

From these 120 dead dogs 34 died unexpectedly and IE was diagnosed in 19 of these 34 dogs. Since 5 of these dogs died during or shortly after status epilepticus, two due to aspiration pneumonia as a result of seizures, seven dogs had to be excluded for further analysis of pSUDED. In summary, in the current study population a total of 12 pSUDED cases were detected (Figure 2), revealing an occurrence of 4.5–10%. The occurrence rate was calculated considering the total population of dogs with IE (12/264, 4.5%) on one hand and dead dogs with IE (12/120, 10%) on the other hand.

Of the 264 dogs evaluated with IE, 63.3% were male (167/264; 104 intact, 63 neutered) and 36.7% female (97/264; 46 intact, 51 neutered), and gender was not found to be a risk factor for pSUDED (Supplementary Table 1).

**Figure 3:**
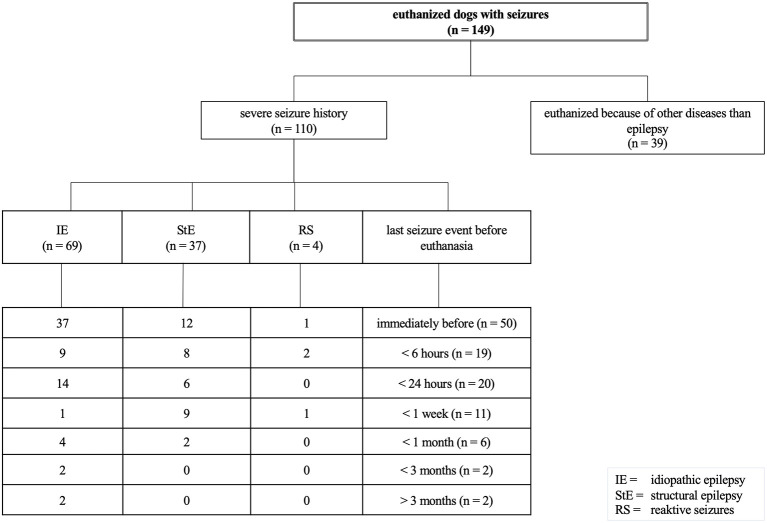
Breed distribution of dogs with idiopathic epilepsy (IE) deceased from probable sudden unexpected death in dogs with epilepsy (pSUDED); A *p* < 0.05 was considered to be significant. The percentage of breeds in the whole study population, dogs with IE, and dogs with pSUDED is presented. Labrador and Golden Retriever were considered as the most represented breeds in this study. Dogs with brachycephalic syndrome were overrepresented (3/12) in the pSUDED group and a odds ratio of 3.68 was calculated.

Ninety different breeds as well as crossbreeds were included, and Labrador Retrievers (21/264), Border Collies, Golden Retrievers (each 15/264 dogs), and Beagles (11/264) were the most frequently presented breeds suffering from IE. However, clustering of BCS dogs (3/12 dogs) in the pSUDED group with an odds ratio of 3.68 was detected. The following distribution of BCS dogs was detected: Chihuahua (*n* = 4), French (*n* = 4) and English Bulldog (*n* = 2), Pug Dog (*n* = 3), Dogue de Bordeaux (*n* = 2), one of each of the following breeds: Boston Terrier, Boxer, Boxer Bulldog Mix, Bulldog Mix, Bullmastiff Boxer Mix, Cavalier King Charles Spaniel, Continental Bulldog, Pekinese and Shih Tzu (24 dogs/264). Comparing dogs with BCS to Labrador and Golden Retrievers, the multivariable regression analysis (Supplementary Table 2) revealed a *p*-value of 0.0015 for dogs with BCS (Figure 3).

The reported frequency of seizures in 10/12 pSUDED cases ranged from more than one seizure per week to one per month. In 2 cases, the seizure frequency ranged from 1 seizure per 2 months to 1 seizure per 6 months. One owner reported his dog was transiently seizure free but developed a phase of high seizure frequency (more than one seizure per week) later on. In the present study, one dog with pSUDED had the last observed seizure within the last month, and two dogs more than 3 months before their death. Multivariable regression analysis displayed no association for seizure frequency on pSUDED (*p* = 0.09).

In the present study, 58.5% (7/12 dogs with pSUDED) had generalized seizures and 41.7% (5/12 dogs with pSUDED) usually had focal seizures with secondary generalization or displayed both. In total, dogs with IE of the study displayed generalized seizures (68.55%; 170/248 dogs), whereas 26.21% (65/248) showed focal seizures turning into generalized seizures or alternating focal and generalized seizures. Only 5.24% (13/248) of owners reported merely focal seizures. The owners of four dogs with IE did not provide information on the seizure phenotype.

In the pSUDED group, 41.7% (5/12) of dogs had already suffered multiple events with status epilepticus and 58.3% (7/12) had displayed multiple cluster seizures. Cluster seizures were a significant finding within the pSUDED group (*p* = 0.0007) and respectively revealed an increased risk for dogs with IE to suffer pSUDED.

Dogs with IE reached an average age of 7.6 years (range 1–17 years). Dogs with pSUDED died at an age of 6 years (mean value; range 2–12 years). Furthermore, it could be determined that dogs with pSUDED had an average lifespan of 3.61 years after the first observed seizure event. The remaining dogs with IE with another cause of death had a survival time of 4.7 years after the initial seizure. It is worth mentioning that the mean survival time of the control dogs with structural epilepsy after the onset of the first seizure event was 3.6 years, of dogs with reactive seizures 4.3 years. On average, dogs with structural epilepsy died at an age of 9.1 years (range 1–17 years) and dogs with reactive seizures 8.2 years (range 1–18 years).

In 2/12 cases with pSUDED death occurred shortly after a seizure event (minutes to hours). Of 12 pSUDED, 7 occurred during the night and 5 during daytime.

**Figure 4:**
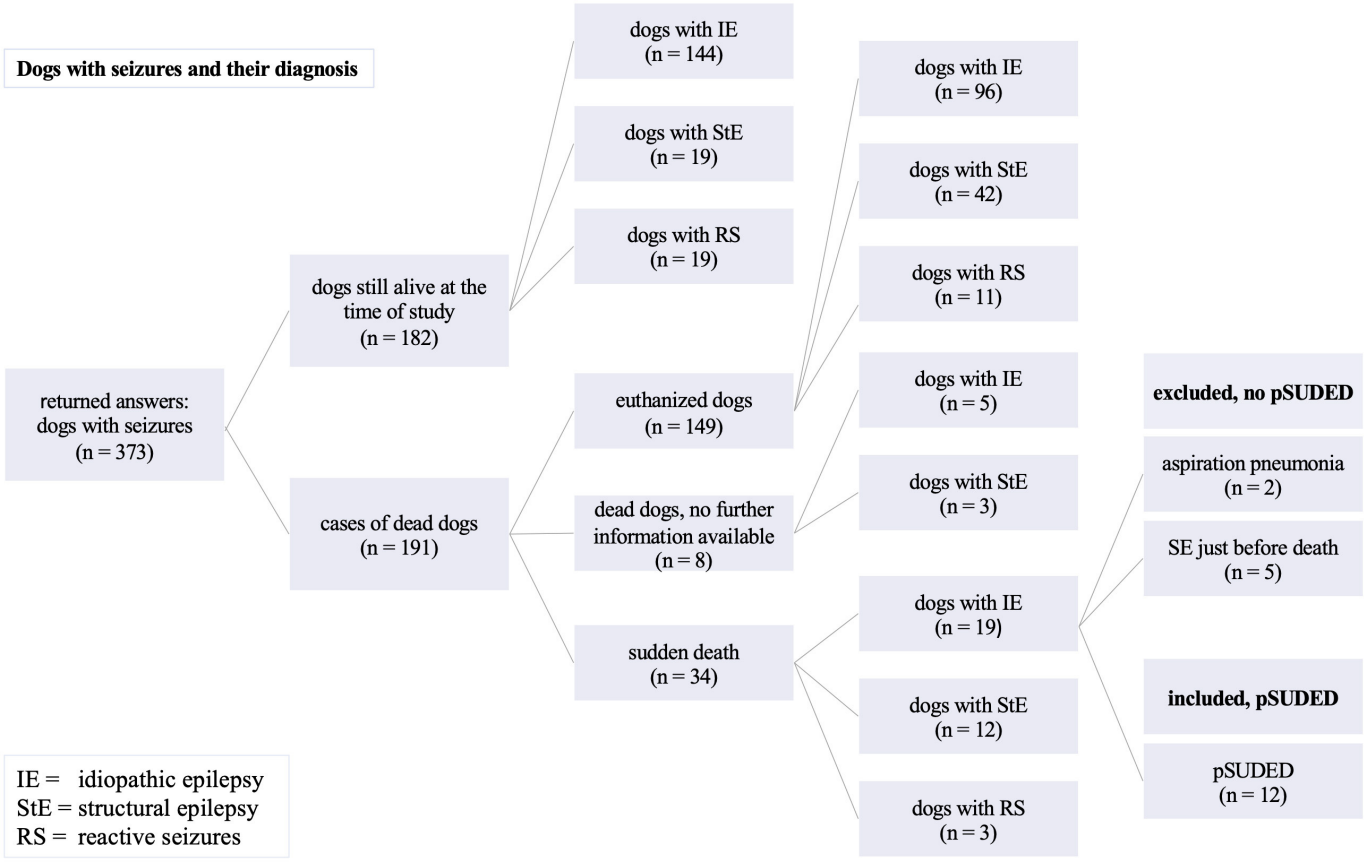
Overview of euthanized dogs with seizures.

To compare pSUDED with other causes of death in dogs with epilepsy or reactive seizures in the current study, the questionnaire was evaluated accordingly. The majority of dead dogs with epilepsy were euthanized because of their disease (110 of 149 euthanized dogs) as shown in Figure 4. Of these 149 euthanized dogs 46% (*n* = 69) were dogs with IE euthanized because of their disease. Of these 69 dogs with IE, 37 showed seizures immediately before euthanasia. Dogs were euthanized because of high seizure frequency or perceived diminished quality of life at a mean age of 7.2 years (range 1–18 years) and thus lived noticeably shorter than dogs euthanized because of other diseases than epilepsy (mean value 10 years, range 2–17 years).

A Chi-square test did not reveal significant difference for the amount of administered AEDs in the pSUDED and the IE group (χ^2^ = 0.72).

## Discussion

Epilepsy is one of the most common neurological diseases presented to the veterinarian (31–33). The risk of sudden death in dogs directly related to status epilepticus or after cluster seizures is well-known (21, 34–36). SUDED has only been presumed in single cases (21–24). The percentage of pSUDED in the current study was 4.5% including 264 dogs with IE, or 10% evaluating only 120 dead dogs with IE. The presented proportional distribution is similar to human medicine reports, where depending on the analysis 7 or 17% of epileptic patients die of SUDEP (3, 5). Whether a dog with IE could die because of pSUDED cannot be predicted with the current state of knowledge. However, it is important to point out that sudden death can occur not only after status epilepticus but ultimately due to epilepsy itself.

There are different approaches to the definitions regarding the inclusion criteria of SUDEP by Nashef et al.: On the one hand, death should occur within 1 h of the last seizure event to be called SUDEP (9). Hence, it should be considered carefully whether a sudden death in dogs can also be classified as pSUDED if the last observed seizure occurred several weeks ago. For this reason, dogs whose last seizure event occurred several weeks ago could have been excluded. However, in a Swedish nationwide population-based case series by Sveinsson et al. with 329 SUDEP cases, SUDEP was actually observed in only 17% of cases and a previous seizure event was often only suspected by indirect signs (*n* = 171) (37). It would be interesting to know if a seizure event preceded death, but accurate observation using 24-h video monitoring was not available in the present study. On the other hand Nashef et al. also described that SUDEP cases as mostly unobserved, especially because “information regarding a terminal seizure occurrence is often limited or unavailable” (9). For this reason, we also include dogs whose last seizure events occurred more than 1 h ago, in order not to pass up a dog.

In the MORTEMUS study, a clinical observational study of SUDEP cases in people, 11 of the 16 SUDEPs showed observed GTCS immediately before death (4). In dogs with IE GTCS is observed more often than focal seizures (36, 38, 39). In comparison, only about 20% of human patients show generalized seizures and about 60% display focal seizures (40, 41). In dogs, it is possible that not all focal seizures are observed (42, 43) which could explain discrepancies between the human and canine species regarding the amount of focal seizures. The seizure frequency with regard to focal seizures may have been underestimated in some cases in this study. However, continuous monitoring throughout the day is rarely possible in private circumstances. In general, the seizure frequency showed no significant difference between the pSUDED group and the remaining dogs with IE (*p* = 0.09), in contrast to some human medicine studies, where the frequency of seizures and generalized tonic-clonic seizures are considered a decisive risk factor for SUDEP (44–46).

In the current study, dogs with pSUDED had multiple events with status epilepticus and cluster seizures in their seizure history. Indeed, an increased risk for the occurrence of pSUDED in dogs with cluster seizures (*p* = 0.0007) was detected and should be considered for treatment adaption and communication with dog owners. In human medicine, status epilepticus is defined as a seizure duration exceeding 30 min (47). This is in contrast to veterinary medicine, where a seizure event that has lasted longer than 5 min or in which consciousness is not fully regained between two seizure events is considered as status epilepticus (26). An increased number of dogs could be affected by pSUDED if only a seizure duration of more than 30 min had been identified as status epilepticus. Thus, dogs that die after a 5-min seizure event could also be declared as pSUDED, enhancing the number of pSUDED cases.

The long survival time of dogs with structural epilepsy can be attributed to the fact that meningoencephalitis cases were also included, which had a correspondingly good life expectancy due to milder forms of the disease. Dogs suffering from pSUDED died at a younger age with a shortened life expectancy by 1.5 years than the other dogs with IE. Gullov et al. showed a similar survival time of IE dogs in their longitudinal observational study (24). Although dogs with IE can reach a similar age to their conspecifics (36), life expectancy in dogs with IE is significantly decreased due to euthanasia on owner's request because of high seizure frequency and in non-responders to therapy (21). This is also shown by the number of euthanized dogs in the current study (46%; 69/149), of which more than 53% (37/69) were euthanized directly after a seizure event. The transfer of one's own well-being or suffering to the dog can influence a decision regarding further treatment or salvation through euthanasia (17, 48). It remains unclear whether the seizure development of these dogs would have led to pSUDED, another factor which could increase the number of pSUDED deceased dogs.

Due to their abnormal anatomy and increasing popularity as companion dogs (49), we were interested if brachycephalic dogs have a potentially increased risk of pSUDED. Indeed, in the present study BCS dogs showed an increased risk for pSUDED (*p* = 0.0015) (Supplementary Table 2). A postictal obstruction due to swelling and accumulation of mucus in the airways is possible in these dogs from an anatomical point of view (18).

In the MORTEMUS study, 14 of 16 SUDEP cases died at night (4). Of the 12 sudden unexpected deaths in the present study in dogs, 7 occurred during the night and 5 during daytime. Kwiatkowska et al. described that during hospitalization 50% of dogs showed another seizure event within 7 h after in house placement (50). Consequently, intensive monitoring during the clinical stay of dogs with IE, especially at night, is absolutely necessary and advisable (51). Six owners were with their dogs when they died. Noteworthy, only three dogs with pSUDED were found already dead by their owners. A recommendation for pet owners with family dogs would be to place the sleeping place of the dog with IE within hearing distance at night (52). Resuscitation can be lifesaving if performed immediately within 3 min after cardiovascular failure (53) and pSUDED might be prevented.

So far, various approaches have been used in studies to describe the pathophysiology of SUDEP. Even though the mechanisms have not yet been fully clarified, SUDEP is assumed to be a multifactorial process. Among other factors, influence is suspected by specific areas in the brainstem, the ascending arousal system (AAS) (54), or ascending reticular activating system (ARAS) (55, 56). Due to the MORTEMUS study and the repetitive pattern of terminal events, the influence of cardiac and respiratory system is also presumed (4, 57–59).

Furthermore, therapy resistant or poorly controlled chronic seizures, GTCS, and a high seizure frequency in human medicine are suspected risk factors (60). Indeed, in the current study, the majority of the 12 pSUDED cases had a high seizure frequency. However, a significant correlation could not be detected for the seizure frequency nor for the number of applied AEDs. Despite the high number of evaluated cases in total, the number of 12 dogs with pSUDED might not be large enough to reach statistical significance in this respect.

Due to the study design, it was not possible to take a closer look at the alignment of the body position of the animals found. In human medicine, people are mainly found in the prone position (4, 12, 61). In contrast, other studies have no or no significant case numbers to report on the body position in SUDEP (62, 63). It is assumed that people during or after a seizure event may not be able to breathe enough and may not change their position in their postictal stage (4, 46, 57, 61).

The number of pathological examinations is noticeably lower in veterinary medicine than in human medicine (64). Only 4.8% of the dog owners at the University of Veterinary Medicine Hannover left their dogs to the Department of Pathology for necropsy (65). Due to the generally low number of pathological examination and because the majority of the dogs died at home, no pathological examination has been recorded in the current cases and only the presence of pSUDED, but not SUDED, could be investigated.

During consultation and briefing of carers with dogs with epilepsy, the information should also include possibility of sudden unexpected death, which could improve their compliance regarding therapy. Strzelczyk et al. illustrated in a survey that only 2.7% of neurologists informed all of their patients about SUDEP (66). However, approximately 90% of people with epilepsy would like to be instructed comprehensively (67). Given the fact that the SMR is increased in human patients with epilepsy than in the general population, the need for education is underlined (2). This evidence from human medical studies underlines the requirement for adequate education in the veterinary field.

The time of investigation and the retrospective nature of the study was chosen to evaluate life expectancy and to observe and evaluate the course of epilepsy over a longer period of time which enabled us to obtain a high number of cases. This study design leads to loss of patient data, because several owners could not be reached per phone call or postal service. Thanks to the initial high number of cases, we were nevertheless able to generate a significant statistical analysis after exclusion of numerous cases. In addition, the cases listed are patients of a University hospital whose owners may have chosen treatment based on the severity of seizures and might therefore not represent the entire canine epilepsy population (35, 68).

Epilepsy is a variable, individual disease process and it is difficult to predict how the frequency and severity of the seizures will vary (39). pSUDED occurs unexpectedly and although there is still concern about the animal's illness, the occurrence of death is not predictable. Nevertheless, an education of the owner about this potential risk as well as management (e.g., sleeping place near the bed, first aid management like resuscitation) should be given to every owner of a dog with IE.

## Conclusions and Clinical Relevance

Life expectancy in dogs with pSUDED is on average 1.5 years shorter than in dogs with IE. The results of this study suggest that dogs with IE but especially those with brachycephalic syndrome and cluster seizures have an increased risk to die of pSUDED. As in human medicine, precautions regarding risk factors may be necessary to keep the number of pSUDED as low as possible. This reinforces the need for early and adequate treatment of seizure events and education.

## Data Availability Statement

Publicly available datasets were analyzed in this study. This data can be found here: https://nbn-resolving.org/urn:nbn:de:gbv:95-114382.

## Ethics Statement

The study was conducted in accordance with the German Animal Welfare Act within the law of animal welfare, following the ethical guidelines of the University of Veterinary Medicine Hannover (approval by the thesis commission and the data protection officer).

## Author Contributions

AT and EH were responsible for the study conception. Data collection had been completed by EH with help of JE. Statistical analysis, data analysis, and manuscript writing were performed by EH. AT supervised data collection and manuscript editing, as well as JN. All authors contributed to the article and approved the submitted version.

## Conflict of Interest

The authors declare that the research was conducted in the absence of any commercial or financial relationships that could be construed as a potential conflict of interest.

## Abbreviations

AED: Antiepileptic drug
BCS: Brachycephalic syndrome
GTCS: Generalized tonic-clonic seizures
IE: Idiopathic epilepsy
(p)SUDED: (Probable) sudden unexpected death in dogs with epilepsy
SMR: Standardized mortality rate
SUDEP: Sudden unexpected death in epileptic patients
TIER I, II: Confidence level for the diagnosis of idiopathic epilepsy.
