# Supplementary material for: Probable Sudden Unexpected Death in Dogs With Epilepsy (pSUDED)
Source: Front Vet Sci. 2021 Apr 27;8:600307. doi: 10.3389/fvets.2021.600307 (PMC8112544; doi:10.3389/fvets.2021.600307)
Supplement: Supplementary file 1 [file Data_Sheet_1.docx]

Supplementary Material

# Supplementary Tables

Table 1 Sex distribution of dogs with idiopathic epilepsy; pSUDED = probable sudden unexpected death in dogs with epilepsy

| sex | male | male neutered | female | female neutered | total |
| --- | --- | --- | --- | --- | --- |
| no pSUDED | 98 | 62 | 43 | 49 | 252 |
| pSUDED | 6 | 1 | 3 | 2 | 12 |
| total | 104 | 63 | 46 | 51 | 264 |

Table 2 Multivariable regression analysis, independent variables: Retriever (Golden and Labrador Retriever) and dogs with brachycephalic syndrome (BCS).

| ANOVA | df | SS | MS | F | Significance F |  |
| --- | --- | --- | --- | --- | --- | --- |
| Regression | 3 | 0,500903998 | 0,166968 | 3,96321899 | 0,00869136 |  |
| Residual | 260 | 10,95364146 | 0,04212939 |  |  |  |
| Total | 263 | 11,45454545 |  |  |  |  |
|  |  |  |  |  |  |  |
|  | Coefficients | Standard Error | t Stat | P-value | Lower 95% | Upper 95% |
| Intercept | 0,0245098 | 0,014370686 | 1,70554167 | 0,08928774 | -0,0037879 | 0,05280755 |
| Golden Retriever | 0,04215686 | 0,054910314 | 0,76774033 | 0,44333815 | -0,0659687 | 0,15028241 |
| BCS breeds | 0,14215686 | 0,044293429 | 3,20943456 | **0,00149699** | 0,05493734 | 0,22937638 |
| Labrador Retriever | 0,07072829 | 0,047039112 | 1,50360601 | 0,13389653 | -0,0218978 | 0,16335442 |

Table 3 multivariable regression analysis, independent variables seizure frequency, breed, CS = Cluster-seizures, SE = Status epilepticus, seizure type (focal, generalized, focal to generalized seizure, focal and generalized seizures).

|  | Coefficients | Standard Error | t Stat | P-value | Lower 95% | Upper 95% |
| --- | --- | --- | --- | --- | --- | --- |
| Intercept | -0.058294166 | 0.054386993 | -1.071840202 | 0.284793398 | -0.165393109 | 0.04880478 |
| seizure frequency | 0.007939916 | 0.004658118 | 1.704532825 | 0.089485433 | -0.001232857 | 0.01711269 |
| breed | 0.131237332 | 0.044487875 | 2.94995727 | **0.003470097** | 0.043631746 | 0.21884292 |
| CS | -0.005753382 | 0.013322406 | -0.431857558 | 0.666205625 | -0.031987882 | 0.02048112 |
| SE | 0.007656132 | 0.011264994 | 0.67963922 | 0.497342248 | -0.01452691 | 0.02983917 |
| seizure type | 0.026228126 | 0017609523 | 1.489428553 | 0.137595948 | -0.008448571 | 0.06090482 |

Table 4 Multivariable regression analysis, independent variables: CS = Cluster-seizures, SE = Status epilepticus.

| ANOVA | df | SS | MS | F | Significance F |  |
| --- | --- | --- | --- | --- | --- | --- |
| Regression | 2 | 74.47963208 | 37.23981604 | 5.787763231 | 0003471771 |  |
| Residual | 261 | 1679.334762 | 6.434232804 |  |  |  |
| Total | 263 | 1753.814394 |  |  |  |  |
|  |  |  |  |  |  |  |
|  | Coefficients | Standard Error | t Stat | P-value | Lower 95% | Upper 95% |
| Intercept | 4.176531272 | 0.499278066 | 8.365140696 | 3.655E-15 | 3.193405466 | 5.15965708 |
| CS | -0.549985708 | 0.161891127 | -3.397256657 | **0.000786883** | -0.868764671 | -0.23120674 |
| SE | -0.048620947 | 0.136818763 | -0.355367537 | 0.722601286 | -0.318030052 | 0.22078816 |

***Supplementary Material***

**1 Supplementary Data**

**Survey – epilepsy/seizures: Life expectancy after epilepsy diagnosis in dogs**

Note

Please tick the appropriate point/-s below or

describe answers in the appropriate line.

*Not word-perfect translation of the questionnaire.*

**Data protection**: declaration according to the Universities rules.

Questions marked with ^(#)^ were taken with minor modifications from the questionnaire of Wessmann et al. (2014 and 2016)
^(#)^ (WESSMANN et al. 2014; WESSMANN et al. 2016).

Questions marked with ^(*)^ had to be answered to continue the online survey.

**2 different pathways in the online questionnaire**

Is your dog still alive? *

- Yes
- No

**Signalment**

Which dog breed is/was affected? _________________________________

Sex of your dog?

- male
- female
- male neutered
- female neutered

Birthday of your dog? _________________________________________

How old is/was your dog? _______________________________________

Weight of your dog?_________________________________

Household, usage of the dog?

- Single animal
- With other animals
- Family dog
- Guard dog
- Service dog/working dog

Did your dog ever suffered a traumatic brain injury?

- Yes
- No
- I don’t know it

If yes, what was the cause?

- Car accident
- Fall
- others ____________________________

Was there any external involvement of the skull/head?

- Yes
- No

How much time passed between trauma and onset of seizures?

- immediately
- within 1 day
- within 1 week
- within 1 month
- within 6 months
- within 1 year
- within 1 – 3 years
- > 3 years
- Seizures already in advance

**Seizures**

Test performed

- General examination
- Blood examination
- Neurological examination
- Examination for hypothyroidism
- X-Ray
- Ultrasound abdomen
- Urine examination
- MRI/magnetic resonance imaging
- CT/computer tomography
- CSF/Cerebrospinal fluid examination
- Other

________________________

What is the diagnosis for your dog?

- idiopathic epilepsy (diagnosis of exclusion, unremarkable examinations)
- structural epilepsy
  - anomaly = mostly congenital malformation

### Explanation of terms:

Idiopathic epilepsy:
unremarkable neurological and clinical examinations and further examinations without further findings

structural epilepsy:
structural abnormalities in the brain, such as infarct, inflammation, trauma, brain tumor, etc.

reactice seizures:
a disease outside the brain, such as intoxication, liver and/or kidney disease, hypoglycemia etc.

- - encephalitis
  - brain tumor
  - Meningoencephalitis
  - Posttraumatic epilepsy = seizures after traumatic brain injury
  - I don’t know it
- reactive seizures
  - Portosystemic Shunt
  - Heart disease
  - Diabetes mellitus
  - Hypocalcemia
  - Hypoglycemia
  - hypothyroidism
  - intoxication
  - I don’t know it
- Genetic cause
- Traumatic brain injury
- I don’t know it

How old was your dog, when the first seizure was observed?

- < 6 months
- 6 - 12 months
- 1 - 3 years
- 4 - 6 years
- > 6 years

How frequently does your dog display seizures?

- > 1 seizure/week
- 1 seizure/week
- 1 seizure/2 weeks
- 1 seizure/month
- 1 seizure/2 months
- 1 seizure/3 months
- 1 seizure/6 months
- Seizure free
- other _______________

Did cluster seizures or Status epilepticus occur?

|  | No | Unique | repeatedly | always |
| --- | --- | --- | --- | --- |
| Cluster seizures | 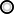 | 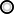 | 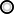 | 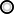 |
| Status epilepticus | 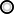 | 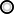 | 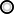 | 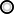 |

### Explanation of terms:

Cluster seizures: more than 2 seizures within 24 hours

Status epilepticus: seizure duration > 5 minutes, or two or more separate seizures without
recovery of consciousness between the seizures

When are seizures observed?

- sleep
- After physical stress
- After stress situations
- I do not know

How did the seizures look like?

- focal
- starting focally, passing into a generalized seizure
- alternating focal and generalized seizures
- tonic
- clonic
- tonic-clonic
- I don’t know it

**Seizure severity and frequency**

### Explanation of terms:

focal: muscle contractions localized to a part of the body, e.g. the face

generalized: whole-body contractions

Please rate the following statements! ^(#)^

|  | Strongly agree | agree | neutral | disagree | Strongly disagree |
| --- | --- | --- | --- | --- | --- |
| In the last 3 months, the frequency of the fits in my dog was acceptable. | 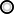 | 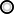 | 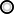 | 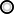 | 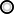 |
| In the last 3 months, the severity of the fits in my dog was acceptable. | 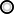 | 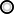 | 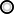 | 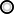 | 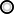 |
| In the last 3 months, overall, the fits  in my dog are  managed successfully. | 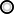 | 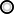 | 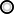 | 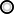 | 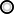 |
|  |  |  |  |  |  |

Overall, how severe were your dog's fits in the past 3 months? ^(#)^

- very mild
- mild
- a moderate amount
- severe
- very severe
- I can’t judge

Carer anxiety around the seizure event and it´s effects on the dog : ^(#)^

|  | Strongly agree | agree | neutral | disagree | Strongly disagree |
| --- | --- | --- | --- | --- | --- |
| In the last 3 months,  I worried about the frequency of the seizures in my dog. | 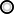 | 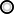 | 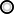 | 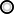 | 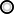 |
| In the last 3 months,  I worried about the severity of the seizures in my dog. | 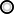 | 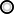 | 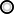 | 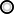 | 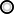 |

**Antiepileptic drugs**

### Explanation of terms:

Antiepileptica = drugs for the treatment of epilepsy and seizures

Have you given your dog antiepileptic drugs after the onset of the disease? *

- Yes
- No

How many antiepileptic drugs do/did you give to your dog daily for seizure control?

- 1
- 2
- 3
- > 3

Which medication(s) are you giving/have you been giving your dog?

- Pexion
- Phenobarbital
- Kaliumbromid
- Levetiracetam
- Zonisamid
- Gabapentin
- Pregabalin
- other: ______________________
- emergency medication
- Diazepam rectal tubes
- Levetiracetam pulse therapy
- other: ___________________

Perception of rectal diazepam tubes ^(#)^

|  | never | rarely | sometimes | usually | always |
| --- | --- | --- | --- | --- | --- |
| Have you ever been uncertain when to  give rectal diazepam? | 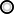 | 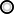 | 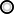 | 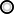 | 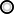 |
| Have you ever been worried how much or how often you are supposed to give rectal diazepam? | 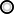 | 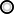 | 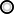 | 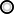 | 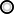 |

Have you noticed any improvement under the medication?

- Yes
- No

In the past 3 months, the side effects of the medication to control the fits in my dog were acceptable. ^(#)^

- No side effects
- Strongly agree
- agree
- neutral
- disagree
- strongly disagree
- I can’t judge

In the past 3 months, how severe was the following side effect: ^(#)^

|  | No side effects | Very mild | mild | A moderate amount | | severe | | Verey severe | | |
| --- | --- | --- | --- | --- | --- | --- | --- | --- | --- | --- |
| Eating more/would like to eat more | 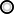 | 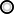 | 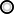 | | 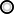 | | 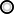 | | 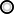 |  |
| Gaining weight | 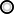 | 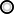 | 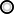 | | 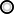 | | 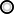 | | 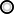 |  |
| Drinking more | 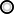 | 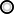 | 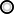 | | 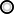 | | 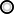 | | 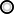 |  |
| Urinating more | 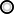 | 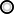 | 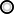 | | 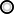 | | 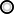 | | 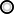 |  |
| Sleeping more | 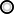 | 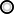 | 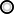 | | 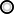 | | 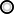 | | 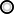 |  |
| Wobbly/not coordinated when walking | 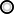 | 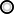 | 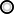 | | 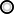 | | 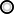 | | 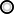 |  |
| Restlessness/pacing | 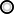 | 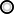 | 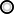 | | 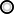 | | 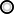 | | 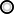 |  |
| Itchiness or skin rash | 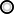 | 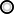 | 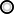 | | 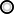 | | 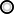 | | 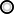 |  |
| Vomiting | 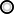 | 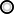 | 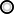 | | 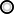 | | 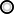 | | 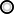 |  |
| Diarrheal | 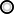 | 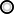 | 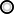 | |  | |  | |  |  |
| Coughing |  |  |  | |  | |  | |  |  |

Carer distaste of antiepileptic drug side effects: ^(#)^

| **How bothersome are:** | Not at all | A little | moderately | Quite a lot | A lot | |
| --- | --- | --- | --- | --- | --- | --- |
| The physical effects of the medication on my dog. |  |  |  |  |  |  |
| The mental effects of the medication on my dog. |  |  |  |  |  |  |

In the past 3 months, how much did you dislike the following side effects? ^(#)^

|  | Not at all | A little | moderately | Quite a lot | A lot |
| --- | --- | --- | --- | --- | --- |
| Eating more/would like to eat more |  |  |  |  |  |
| Gaining weight |  |  |  |  |  |
| Drinking more |  |  |  |  |  |
| Urinating more |  |  |  |  |  |
| Sleeping more |  |  |  |  |  |
| Wobbly/not coordinated when walking |  |  |  |  |  |
| Restlessness/pacing |  |  |  |  |  |
| Coughing |  |  |  |  |  |

**Social aspects of the carer and dog**

Restrictions on the carer’s life (related to caring for a dog with epilepsy/seizures): ^(#)^

|  | never | Not often | Sometimes | Often | Very often |
| --- | --- | --- | --- | --- | --- |
| In the past 3 months, how often did you feel that your dog’s epilepsy caused conflict with your work, education or day-to-day activities? |  |  |  |  |  |
| In the past 3 months, how often did you feel that your dog’s epilepsy limited your social life? |  |  |  |  |  |
| In the past 3 months, how often did you feel that your dog’s epilepsy limited your independence? |  |  |  |  |  |

Frustrations over caring for a dog with epilepsy/seizures: ^(#)^

| **How bothersome are** | Not at all | A little | moderately | Quite a lot | A lot |
| --- | --- | --- | --- | --- | --- |
| My limitations in work, education or day-to-day activities because of my dog’s seizures? |  |  |  |  |  |
| My social limitatios because of my dog’s seizures? |  |  |  |  |  |
| Overall, my limitations on my life caring for my epileptic dog? |  |  |  |  |  |

How serious do you/did you perceive the side effects of antiepileptic drugs on your dog's quality of life?

- No side effects
- very mild
- mild
- a moderate amount
- severe
- very severe
- I can’t judge

The following pages are addressed to you if your dog unfortunately is no longer alive.**Cause of death**

Did your dog die or was he euthanised?

- My dog died on its own
- My dog was euthanized

If your dog has been put down, please do not answer the questions until the next page.

When was the last seizure event before your dog died?

- Just before death
- Less than 6 hours before death
- Less than 24 hours before death
- Less than 1 week before death
- Less than 1 month before death
- Less than 3 months before death
- More than 3 months before death

Time of the day, when your dog died?

- During the day
- At night

Could you be with your dog when he died or did you find him dead?

- I was with him
- I have found him dead
- other ___________________________

Was the passing away sudden and unexpected for you?

- Yes
- No

Did your dog die during a seizure?

- Yes
- No

seizure duration?

### Explanation of terms:

Cluster seizures: more than 2 seizures within 24 hours

Status epilepticus: seizure duration > 5 minutes, or two or more separate seizures without recovery of full consciousness between seizures

- Less than 2 minutes
- Cluster seizures
- Status epilepticus
- I am not in a position to judge
- remarks

_______________

When was the last seizure event before your dog was euthanised?

- Just before death
- Less than 6 hours
  before death
- Less than 24 hours
  before death
- Less than 1 week
  before death
- Less than 1 month
  before death
- Less than 3 months
  before death
- More than 3 months
  before death

Has your dog been euthanised because of epilepsy or because of any of the following diseases?

- Yes, because of epilepsy
  - high frequency of seizures despite medication
  - too severe side effects of antiepileptic drugs and seizures
  - in status epilepticus
  - Cluster seizures
  - Financial reasons
  - Too severe impairment of the quality of life of my dog
  - Too severe impairment of my personal quality of life
  - other: _______________________________________________
- No, he was euthanised because of age or one of the following diseases
  - neoplasia
  - orthopedic disease
  - heart disease
  - behavioural abnormalities
  - (car) accident
  - Gastrointestinal tract problems
  - skin disease
  - other: ________________________________________________
- Among other things also because of the epilepsy

Thank you very much for participating and answering this survey. In this way you help many dog owners in the same situation. Our goal is to improve therapies as needed and to provide advice to the dog owner.

^#)^

WESSMANN, A., H. A. VOLK, R. M. PACKER, M. ORTEGA u. T. J. ANDERSON (2016):

Quality-of-life aspects in idiopathic epilepsy in dogs.

The Veterinary record 179, 229

WESSMANN, A., H. A. VOLK, T. PARKIN, M. ORTEGA u. T. J. ANDERSON (2014):

Evaluation of quality of life in dogs with idiopathic epilepsy.

Journal of veterinary internal medicine 28, 510-514
